# Association of *CFH* and *CFB* Gene Polymorphisms with Retinopathy in Type 2 Diabetic Patients

**DOI:** 10.1155/2013/748435

**Published:** 2013-06-24

**Authors:** Jun Wang, Ming Ming Yang, Yan Bo Li, Guo Dong Liu, Yan Teng, Xiao Min Liu

**Affiliations:** ^1^Department of Endocrinology, First Affiliated Hospital of Harbin Medical University, 23 Post Road, Nangang Region, Harbin, Heilongjiang 150001, China; ^2^Eye Hospital, First Affiliated Hospital, Harbin Medical University, 23 Post Road, Nangang Region, Harbin, Heilongjiang 150001, China

## Abstract

*Objectives.* The complement system is a key component of innate immunity and has been implicated in the pathogenesis of diabetic retinopathy (DR). This study aimed at investigating whether polymorphisms of two genes in the complement pathway, complement factor H (CFH) and complement factor B (CFB), are associated with DR. *Methods.* 552 well-defined subjects with type 2 diabetes, consisting of 277 DR patients and 275 diabetic controls, were recruited. Four Tag-SNPs rs1048709, rs537160, rs4151657, and rs2072633 in CFB and rs800292 (I62V) in CFH were examined using TaqMan Genotyping Assays. *Results.* There were significant increases in the frequencies of A allele and AA genotype for rs1048709 in DR patients compared with diabetic controls (*P*_corr_ = 0.035, OR = 1.42; *P*_corr_ = 0.02, OR = 2.27, resp.): meanwhile, significant decreases in the frequencies of A allele and AA genotype for rs800292 were observed in DR patients compared with diabetic controls (*P*_corr_ = 0.04, OR = 0.72; *P*_corr_ = 0.015, OR = 0.51, resp.). Joint effect of these two loci was also identified. Moreover, rs800292/AA genotype was found to be related with delayed progression to DR. *Conclusions.* CFH-rs800292 and CFB-rs1048709 are associated with the presence of DR, which strengthens the concept that complement system plays an important role in the pathogenesis of DR.

## 1. Introduction

The prevalence of diabetes has been reaching epidemic proportions at an alarming rate currently. Diabetic retinopathy (DR) is the most common microvascular complication of diabetes and is a leading cause of blindness worldwide, characterized by increased vascular permeability, tissue ischemia, and neovascularization [1, 2]. To date, many environmental and clinical factors have been proposed to affect the development of DR, such as alteration of glucose metabolism, poor glycemic control, and prolonged duration of diabetes [3, 4]. In addition, an important conceptual consideration is that DR can manifest in individuals with genetic predisposition through the existence of familial aggregation of severe DR among the siblings and family member of diabetic patients [5–7]. The pathogenesis of DR is complex and has multifactorial causes. Several molecules and metabolic pathways like oxidative stress, upregulation of growth factors, activation of protein kinase C (PKC) pathway, and so forth have been implicated in the pathogenesis of DR [8–10]. Recent research insights describing DR as a retinal disease associated with inflammation have drawn special attention and garnered great research interests, and the evidence comes from the observation of typical features such as tissue edema, increased leukostasis, upregulation of inflammatory mediators, and complement activation [11–13]. In view of such inferences, many inflammatory molecules are being investigated as a target for a possible remedy in DR.

The complement system is a key component of innate immunity, which can be divided into the classic, lectin, and alternative pathways and is involved in modulating various immune and inflammatory responses [14, 15]. Under normal conditions, the complement system is continuously active at a low level and is tightly regulated by complement regulators. Disruption in the balance of complement activation and regulation will result in harmful effects and can contribute to various inflammatory diseases, such as age-related macular degeneration (AMD), systemic lupus erythematosus (SLE), rheumatoid arthritis (RA), and Alzheimer's disease [16–20]. Increasing evidence from *in vitro* and *in vivo* studies suggests a pathogenetic role of the complement system in the development of diabetic angiopathy. In these studies, increased expression of several complement factors, namely, complement factor H (CFH), complement factor B (CFB), component 3 (C3), and component 5 (C5), has been observed in the vitreous of DR patients [21–23]. In addition, genetic variants in the *CFH* and *CFB* genes have been also shown to be associated with a range of inflammatory diseases [24–27]. Among the various polymorphisms in the *CFH* gene, I62V (rs800292) was actively investigated and showed strong association with AMD, a disorder shares many pathophysiological features in common with DR, and both appear to involve the inflammation and complement activation. Moreover, functional analyses revealed that rs800292 was associated with levels of complement proteins in serum, and this functional variant was also found to affect the protein-binding affinity with C3b and subsequently reduced the activation of complement alternative pathway. CFB, an opponent of *CFH*, involved in the alternative pathway with the same binding site of C3b, also contributes to regulate the activation of complement cascade, and the genetic impact of *CFB* on DR is of interest [28]. 

Therefore, the purpose of the present study was to test a possible association of *CFH* variant, rs800292 (I62V), and four common variants of *CFB* gene by tag SNP selection with susceptibility to DR. Moreover, since the clinical features and causes of DR are variable, we also evaluated the genotype-phenotype correlations to identify factors associated with prognosis and risk stratification.

## 2. Methods

### 2.1. Study Design and Subjects

The study involved 552 unrelated individuals with type 2 diabetes mellitus (DM) with a defined ophthalmologic status, who were recruited from the First Affiliated Hospital of Harbin Medical University, Harbin, China. Diagnosis of type 2 diabetes was based on World Health Organization criteria [29]. The study was approved by the Bioethics Committee of the Harbin Medical University. All the procedures were conducted according to the tenets of the Declaration of Helsinki. Informed consent was obtained from all study subjects after explanation of the nature of the study.

Ocular examination was performed by independent ophthalmologist using fundus ophthalmoscopy after pupil dilatation. The stage of DR was determined according to the Early Treatment Diabetic Retinopathy Study (ETDRS) criteria [30]. Of this group, 277 patients with type 2 diabetes were diagnosed with DR: 171 (61.7%) with nonproliferative DR (NPDR) and 106 (38.3%) with proliferative DR (PDR). The control group consisted of 275 subjects without DR but with type 2 diabetes duration of more than 10 years. All subjects underwent a detailed ophthalmologic examination and clinical information collection, including corrected visual acuity, fundoscopic examination, age, gender, duration of diabetes and DR, body mass index (BMI), HbA1c level, smoking status, presence of hypertension and hyperlipidemia, and treatment details. People with any of the following situations were excluded from the study: peripheral vascular diseases, acute infection, systemic inflammation diseases, or any other ocular disorders such as AMD, glaucoma, or branch retinal venous occlusion. Overt diabetic nephropathy patients were excluded, the exclusion criteria were as follows: microalbumin creatinine ratio >30 mg/g and urinary microalbumin level >300 mg/d [31].

### 2.2. DNA Extraction and Genotyping

Genomic DNA was extracted from peripheral blood with the QIAamp Blood kit (Qiagen, Hilden, Germany) according to the supplier's instructions. One *CFH* variant, rs800292 (I62V), and four tagging-SNPs (rs1048709, rs537160, rs4151657 and rs2072633) captured 100% of alleles in the *CFB* locus with MAF larger than 0.1, and a mean *r* ^2^ of 1.0 were selected. All the SNPs were genotyped by *Taq*Man SNP Genotyping Assays (Applied Biosystems Inc., Foster City, CA, USA) in the Light Cycler 480 Genotyping Master (Roche Diagnostics Inc., Mannhein, Germany) according to manufacturers' protocols. The PCR amplifications were performed with the thermal cycling conditions of 94°C for 10 min, followed by 40 cycles of 94°C for 15 s, and 60°C for 1.5 min. The genotypes were read by Prism 7000 SDS software (version 1.1; ABI).

### 2.3. Statistical Analysis

Hardy-Weinberg equilibrium (HWE) for genotype frequencies of the SNPs was tested by **χ** ^2^ test. Allelic and genotypic frequencies between DR and DM were compared by **χ** ^2^ test or Fisher exact test. Dominant and recessive models were also applied to investigate the disease association with regard to the minor allele (rs800292[A], rs1048709[A], rs537160[A], rs4151657[C] and, rs2072633[G]). Stratified analysis based on DR severity (NPDR and PDR) was also performed. Logistic regression analysis was applied to adjust the association of these SNPs with age and gender. The Student *t* test and **χ** ^2^ test were used to compare continuous clinical data and categorical variables, respectively. Pairwise linkage disequilibrium (LD, *D*′) between polymorphisms and EM-based haplotype association analysis was performed by Haploview (ver. 4.2). Odds ratios (OR) and 95% confidence intervals (CI) were also calculated. *P* < 0.05 was considered as statistically significant. *P* values were corrected by Bonferroni test for multiple comparisons (*n* = total number of SNPs). The correction for multiple testing in the haplotype analysis was performed by permutation testing.

## 3. Results

### 3.1. Study Group Comparison

The demographic and clinical characterization of the study subjects is presented in Table 1. DM control group had a longer duration of type 2 diabetes compared to DR group (*P* < 0.001). Additionally, DR group had a higher proportion of hyperlipidemia and higher prevalence of insulin therapy than the DM controls (*P* < 0.01 and *P* < 0.001, resp.). There was no significant difference in age, gender, HbA1c level, BMI, proportion of hypertension, smoking status, and family history of diabetes. 

### 3.2. Association Analysis

All genotype frequencies of the five selected SNPs followed the Hardy-Weinberg equilibrium in all subjects. Regarding *CFH* rs800292, there was a significant decrease in the frequencies of A allele and AA homozygosity in DR patients compared with DM controls (*P*_corr_ = 0.04, OR = 0.72, 95% CI = 0.57–0.92; *P*_corr_ = 0.015, OR = 0.51, 95% CI = 0.33–0.80 resp.), indicating a protective effect. Meanwhile, significant association was also detected at *CFB* rs1048709, where there was a significant increase in the frequencies of A allele and AA homozygosity in DR patients compared with DM controls (*P*_corr_ = 0.035, OR = 1.42, 95% CI = 1.10–1.83; *P*_corr_ = 0.02, OR = 2.27, 95% CI = 1.28–4.04 resp.). No significant differences in the genotypic or allelic frequencies were observed for other three SNPs between DR and DM after multiple testing correction (Table 2). In addition, logistic regression analysis was used to assess the role of gene polymorphisms in DR after adjustment for age, gender, duration of diabetes, hyperlipidemia, and insulin therapy, the results showed that the association did not alter between DR and all the 5 SNPs after adjusting for these factors (data not shown).

### 3.3. Linkage Disequilibrium and Haplotype Association Analysis

**Figure 1:**
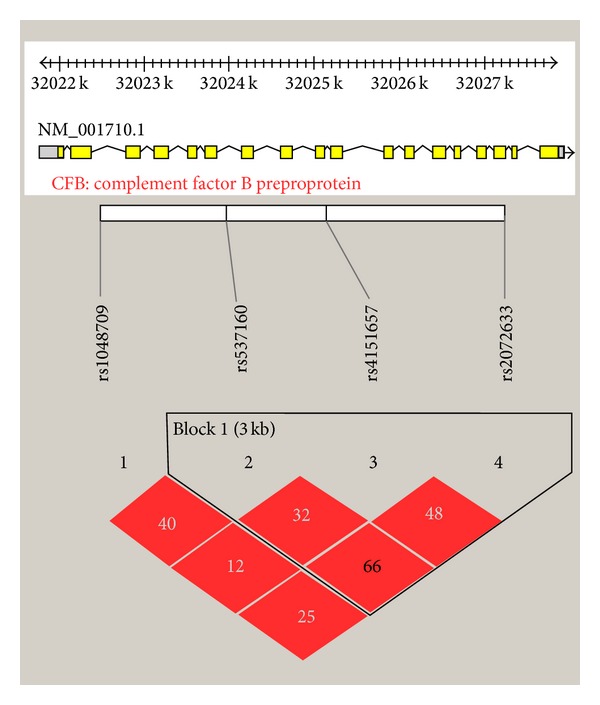
Pairwise LD among three SNPs in *CFB* gene. Linkage disequilibrium was measured by the *D*′ statistic using the data from all subjects. A *D*′ value of 100 indicates a complete LD between 2 markers, and a *D*′ value of 0 indicates a complete linkage equilibrium. Haplotype version 4.2 software was used.

Pairwise LD analysis was performed across the *CFB* locus by using these 4 SNPs, and one haplotype block was detected including 3 SNPs in *CFB* (rs537160, rs4151657, and rs2072633; Figure 1). A protective haplotype, GTG, defined by these 3 SNPs was identified, and conferred a 1.45-fold significantly decreased risk of DR, but the statistical significance would not remain after correction for (*P* = 0.028, permutation *P* = 0.11; Table 3).

### 3.4. Genotype-Phenotype Correlation Analysis

**Figure 2:**
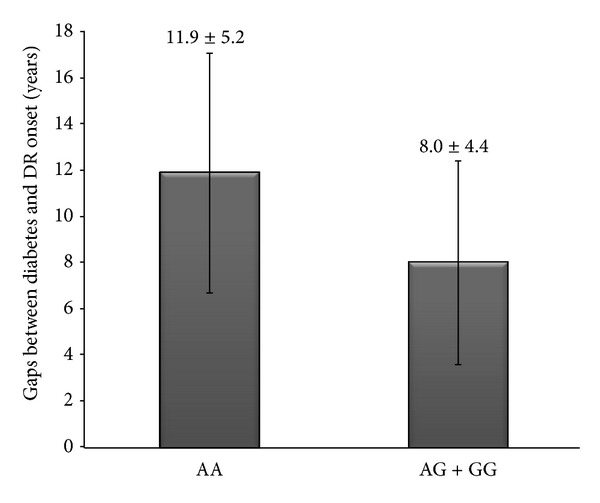
Comparison of gaps between diabetes and DR onset (years) in two genotype groups for *CFH* rs800292 in DR patients (*P* < 0.001).

Among the 277 DR patients, 171 (61.7%) were NPDR and 106 (38.3%) were PDR. Further stratification comparison was also performed on the clinical severity in terms of DR scale. Between NPDR and PDR groups, no significant differences were detected in the allelic and genotypic frequencies of all 5 SNPs (data not shown). Given the significance of *CFH* rs800292 and *CFB* rs1048709, in this study, as well as the observation of recessive effect, correlations of their genotype group (homozygous minor allele versus major allele carriers) with clinical features were evaluated. The results demonstrated that DR patients carrying protective rs800292/AA genotype would present a longer gap (years) between diabetes and DR onset compared with that in patients carrying AG + GG (11.9 ± 5.2 versus 8.0 ± 4.4, *P* < 0.001; Figure 2), and such difference was not observed for *CFB* rs1048709 (9.6 ± 5.6 versus 8.3 ± 4.5, *P* = 0.11). No significant difference was detected in other clinical features between different genotype groups (data not shown). 

### 3.5. Joint-Effect Analysis

**Figure 3:**
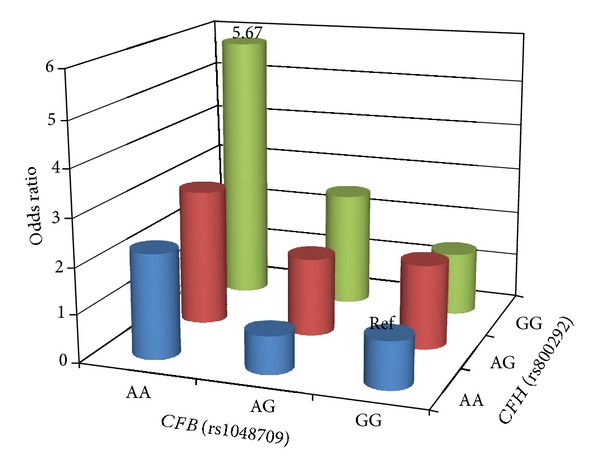
Two loci of *CFH* and *CFB* genotype-specific DR risk.

Considering the biological relevance of CFH and CFB, combined effects of rs800292 and rs1048709 were assessed, and the corresponding ORs of DR for each possible combination of the genotypes of the two loci were estimated (Tables 4(a) and 4(b)). The ORs were compared with the baseline genotype of the two genes. The frequency of the homozygous risk genotypes at both loci was 3.3-fold higher in DR (6.1%) than in DM controls (1.8%) (*P* = 0.002; Table 4(a)). A joint OR of 5.67 in individuals with both homozygous risk alleles was observed (Figure 3).

## 4. Discussion

In this study, we investigated the association of complement genes in type 2 diabetes patients with DR. Our results demonstrated that *CFH *rs800292 (I62V) and *CFB* rs1048709 (R150R) were significantly associated with the susceptibility of developing DR. Moreover, since significant differences have been observed between DM and DR group (duration of diabetes, level of hyperlipidemia, and percentage of insulin therapy), multivariate logistic regression analysis was performed to assess the genetic role of DR by considering these factors, which showed that I62V and R150R remain significant when adjusted for all these factors, implying that *CFH* and *CFB* polymorphisms are independent genetic factors for susceptibility to DR. Over recent years, great advances have been made in understanding the genetic background of the disease; more than 30 candidate genes involved in different metabolic mechanisms and functional pathways have been reported, while, few of them were found to have a strong association [32, 33]. Our findings provide additional and convinced evidence for the involvement of complement system in relation to DR. To our knowledge, the genetic associations of variants in *CFH* and *CFB* with DR have not been described previously.

Activated complement is a “double-edged sword” which might cause self-tissue damage especially for sensitive organs like the eyes. The *CFH* gene is located in chromosome 1 (1q32), which is a major soluble inhibitor of the alternative pathway for controlling complement activation [34]. *CFH* rs800292 has been found to be associated with many inflammatory and neovascular diseases, and the change of rs800292 G > A nucleotide results in the synthesis of Isoleucine instead of Valine. This might leads to structural changes affecting the ability of C3b binding and reducing the activation of the alternative pathway. This subsequently causes excessive activation of the complement system to induce inflammatory disorders [28]. *CFB* gene is located tandemly in the major histocompatibility complex (MHC) class III region, a cluster on chromosome 6p21 with respect to inflammation [35]. As mentioned above, CFB is a competitor of CFH, both involved in the complement alternative pathway. Conceivably, much like impaired CFH-mediated complement inhibition confers DR risk, decreased complement activation by CFB might also serve to affect DR. Not surprisingly, a joint effect of *CFH* and *CFB* risk homozygosity with an OR of 5.67 was identified in this study. In addition, *CFB* polymorphisms were also found to be associated with other inflammatory diseases, such as AMD, lupus, and atypical Hemolytic-uremic syndrome (aHUS) [26, 27, 36]. Furthermore, *in vivo* study has revealed that human RPE cells can synthesize and express CFB and CFH, and the level of CFB was increased in the vitreous of PDR patients [37, 38]. These findings further strengthen the concept that complement system, especially the alternative pathway, plays an important role in the pathogenesis of DR. Unfortunately, the exactly pathogenic significance of the association of *CFH* and *CFB* polymorphisms with DR remains unclear; in this study, these variants in *CFB* represent either synonymous substitutions (rs1048709 R150R) or intronic SNPs, and there is no information on its biological functions currently. One possible explanation is that these polymorphisms may be linked with an undiscovered but biologically relevant structural variant in this region; alternatively, synonymous or intronic regulation could be involved in gene transcription or tissue specificity of gene expression. Further investigations of this region by extensive sequencing to uncover unknown variation are therefore requested.

In the genotype-phenotype analysis, our findings demonstrated a significant relationship between *CFH* rs800292 and duration (in years) between DM and DR onset the protective AA genotype showed association with delayed progression of DR; however, it needs further corroboration by considering factors such as duration of diabetes and glycemic control. Nevertheless, the results not only extend the genetic spectrum of DR, but also provide novel understanding for the genetic impact on disease prognosis. Stratification analysis by DR scale showed that there were no significant differences in the allelic and genotypic frequencies for all 5 SNPs between PDR and NPDR groups, implying that genetic variations of *CFH* and *CFB* might not be associated with DR severity. 

The strength of our study is that all patients and controls are of the same ethnic origin. All subjects were examined in a predetermined standardized order, with strict diagnostic criteria. Moreover, to our best knowledge, this is the first genetic study to investigate the associations of complement factor genes in DR patients. However, there are certain limitations of this study which includes the relatively small sample size, thus, nonsignificant SNPs in the *CFB* might lack adequate power. Secondly, it is a retrospective case-control study that thus lacked the details of follow-up information. Finally, the SNPs selected in our study might not fully reflect the disease risk of unexamined variants in the genes. Further evaluation of these genes by direct sequencing to uncover more variants will be beneficial to identify variants with relevant function in DR. 

In summary, this study first revealed that *CFH* and *CFB* polymorphisms are associated with the development of DR, as well as with delayed progression to DR in type 2 diabetes. A joint effect between *CFH* rs800292 and *CFB *rs1048709 conferring a significantly increased risk for DR was also identified. Further studies to replicate these candidate SNPs in others ethnic groups and determine the biological roles of these polymorphisms in DR are worthwhile.

## Figures and Tables

**Table 1:** Demographic and clinical characteristics of the study subjects.

Characteristic	DR (*n* = 277)	DM (*n* = 275)	*P* value
Age (years)	55.5 ± 14.0	56.3 ± 7.6	0.44
Gender (F/M)	144/133	153/122	0.39
Duration of diabetes (years)	13.6 ± 7.1	18.1 ± 6.7	<0.001
Duration of DR (years)	5.0 ± 4.2	none	
Gaps between diabetes and DR onset (years)	8.3 ± 6.7	none	
HbA1C (%)	8.2 ± 1.7	7.9 ± 1.9	0.09
BMI (kg/m^2^)	23.7 ± 4.6	24.1 ± 4.4	0.46
Hypertension (%)	71.8	66.9	0.21
Hyperlipidemia (%)	32.1	23.3	<0.01
Smoking (%)	13.4	16.0	0.38
Insulin therapy (%)	45.1	26.2	<0.001
Family history of diabetes (%)	25.6	21.5	0.25

All *P* values were compared by *χ* ^2^ or student *t*-test, *P* < 0.05 as statistically significant.

DR: diabetic retinopathy; DM: diabetes mellitus; HbA1c: glycosylated hemoglobin; BMI: body mass index.

**Table 2:** Genotype and allele frequencies of *CFH* and *CFB* polymorphisms in DR patients and DM controls.

SNP ID	Designation	Allele distribution (%)			*P* value	Odds ratio	Genotype distribution (%)			*P* value	Odds ratio
		DR (*n* = 554)		DM (*n* = 550)	(*P*_corr_)	(95% CI)	DR (*n* = 277)		DM (*n* = 275)	(*P*_corr_)	(95% CI)
*CFH *											
rs800292	G > A	A	208 (37.5)	250 (45.5)	0.008 (0.04)	0.72 (0.57–0.92)	AA	38 (13.7)	65 (23.6)	0.15*	0.77 (0.55–1.10)
	Exon2 (I62V)	G	346 (62.5)	300 (54.4)			AG	132 (47.7)	120 (43.6)	0.003^†^ (0.015)	0.51 (0.33–0.80)
							GG	107 (38.6)	90 (32.7)		

*CFB *											
rs1048709	G > A	A	197 (35.6)	154 (28.0)	0.007 (0.035)	1.42 (1.10–1.83)	AA	40 (14.4)	19 (6.9)	0.07*	1.36 (0.97–1.90)
	Exon3 (R150R)	G	357 (64.4)	396 (72.0)			AG	117 (42.2)	116 (42.2)	0.004^†^ (0.02)	2.27 (1.28–4.04)
							GG	120 (43.3)	140 (50.9)		
rs537160	G > A	A	268 (48.4)	266 (48.4)	1.0	1.0 (0.79–1.27)	AA	62 (22.4)	60 (21.8)	0.88*	0.97 (0.66–1.43)
	IVS7	G	286 (51.6)	284 (51.6)			AG	144 (52.0)	146 (53.1)	0.87^†^	1.03 (0.69–1.55)
							GG	71 (25.6)	69 (25.1)		
rs4151657	T > C	C	149 (26.9)	138 (25.1)	0.49	1.10 (0.84–1.44)	CC	21 (7.6)	14 (5.1)	0.79*	1.05 (0.75–1.46)
	IVS10	T	405 (73.1)	412 (74.9)			CT	107 (38.6)	110 (40.0)	0.23^†^	1.53 (0.76–3.07)
							TT	149 (53.8)	151 (54.9)		
rs2072633	A > G	G	330 (59.6)	311 (56.5)	0.31	1.13 (0.89–1.44)	GG	93 (33.6)	86 (31.3)	0.23*	1.32 (0.84–2.07)
	IVS17	A	224 (40.4)	239 (43.5)			AG	144 (52.0)	139 (50.5)	0.56^†^	1.11 (0.78–1.59)
							AA	40 (14.4)	50 (18.2)		

Data analysis was performed by *χ* ^2^ test.

*P value for dominant model.

^†^ *P* value for recessive model.

**Table 3:** Haplotype analysis of *CFB* Polymorphisms between DR and DM.

Haplotype	Frequency	Frequency		*P*	*P*_corr_	Odds ratio 95% CI
		DR	DM			
ATA	0.482	0.482	0.482	0.99	NS	—
GCG	0.260	0.269	0.251	0.49	NS	—
GTG	0.157	0.133	0.182	0.028	NS	0.69 (0.50–0.96)
GTA	0.099	0.114	0.084	0.095	NS	—

*P*_corr_ association analysis results from permutation test (iterations, 10,000).

**Table:** (a)

Genotype distribution	*CFH* rs800292					
*CFB* rs1048709	DM (*n* = 275)			DR (*n* = 277)		
	AA	AG	GG	AA	AG	GG
GG	35 (12.7)	53 (19.3)	52 (18.9)	21 (7.6)	57 (20.6)	42 (15.2)
AG	27 (9.8)	56 (20.4)	33 (12.0)	13 (4.7)	56 (20.2)	48 (17.3)
AA	3 (1.1)	11 (4.0)	5 (1.8)	4 (1.4)	19 (6.9)	17 (6.1)

**Table:** (b)

Joint odds ratios and 95% confidence	*CFH* rs800292		
*CFB* rs1048709	AA	AG	GG
GG	1.00 (Ref)	1.79 (0.93–3.46)	1.35 (0.68–2.65)
AG	0.80 (0.34–1.89)	1.67 (0.87–3.21)	2.42 (1.20–4.88)
AA	2.22 (0.45–10.92)	2.88 (1.65–7.22)	5.67 (1.82–17.62)
